# Serum soluble fas ligand levels and peripheral blood lymphocyte subtypes in patients with drug induced maculopapular rashes, dress and viral exanthemas

**DOI:** 10.1186/1939-4551-8-S1-A182

**Published:** 2015-04-08

**Authors:** Mehtap Yazicioglu, Burhan Turgut, Pinar Gokmirza Ozdemir

**Affiliations:** 1 Trakya University, Turkey

## Background

Fas/Fas ligand (FasL)-dependent apoptotic pathway was reported to be involved in the pathogenesis of drug induced maculopapular rashes (MPRs). In this study, we investigated serum soluble FasL level to discriminate drug-induced skin reactions from other clinically resembling skin diseases such as exanthematous viral infections. We also eveluated the role of T cells in various drug-induced diseases.

## Methods

We analyzed 7 patients with drug induced MPRs (group I), 17 patients with viral exanthemas (group II), 6 paients with DRESS [grup III], and 15 healthy children with no history of adverse drug reactions. A complete blood count and immunophenotyping of peripheral blood lymphocytes were carried out, as well serum FasL levels were analyzed in group I-III (Human sFas-L ELISA kit, eBioscience, Vienna, Austria), within 2 days after the onset of the skin eruptions. Tests were repeated between days 3-5 and days 6-10. In group IV, these analyses were performed once. Liver and renal functions were also eveluated in group I-III. Serum immunoglobulin levels were analyzed in group 3. Skin tests with the suspected drug were applied in cases in group I and III according to the guidelines. In group II, skin tests, drug provocation tests, and viral serology were performed if needed.

## Results

Absolute numbers of peripheral blood lymphocytes ans sFasL levels in initial samples of cases in 4 groups are summarized in Table 1.

**Table 1:** 

		Group 1	Group 2	Group 3	Group 4
sFasL (ng/ml)	Mean±SD	0.20±0.14	0.24±0.16	0.22±0.16	0.19±0.21

	Median	0.27	0.20	0.18	0.10

CD3+/CD4+ cells*	Mean±SD	1508±1230	1761±824	918±552	1240±347

	Median	1026	1604	741	1212

CD3+/CD8+ cells*	Mean±SD	1735±1839	888±463	509±355	723±252

	Median	1376	910	407	624

CD19+ cells*	Mean±SD	622±322	1049±659	195±181	535±286

	Median	630	834	132	489

CD3)/CD16+/CD56+(NK cells)*	Mean±SD	151-980	264-3009	20-487	103-1241

	Median	250±238	181±111	94±34	253±235

*Counts, cells/ml

B cell counts were low in group III when compared to group I and IV. CD4+cells, CD19+cells and NK cells were low in group III when compared to group II. There were no significant differences in sFasL levels between the groups.

## Conclusions

In our study, sFasL levels were not found to be useful to discriminate viral exanthemas from drug rashes. Additionally, the results were not found to be different on repeated evaluations. The only significant difference between drug induced MPRs and DRESS was B cell counts. The low numbers of B cells in DRESS within the first 2 days of the symptoms might be a useful predictor of DRESS development.

